# Arbuscular mycorrhizal symbioses alleviating salt stress in maize is associated with a decline in root-to-leaf gradient of Na^+^/K^+^ ratio

**DOI:** 10.1186/s12870-021-03237-6

**Published:** 2021-10-07

**Authors:** Hao Wang, Tingting An, Di Huang, Runjin Liu, Bingcheng Xu, Suiqi Zhang, Xiping Deng, Kadambot H. M. Siddique, Yinglong Chen

**Affiliations:** 1 State Key Laboratory of Soil Erosion and Dryland Farming on the Loess Plateau, Institute of Soil and Water Conservation, Chinese Academy of Sciences, and Northwest A&F University, Yangling, Shaanxi 712100 China; 2 University of Chinese Academy of Sciences, Beijing, 100049 China; 3 Institute of Mycorrhizal Biotechnology, Qingdao Agricultural University, Qingdao, Shandong 266109 China; 4 The UWA Institute of Agriculture, & School of Agriculture and Environment, The University of Western Australia, Perth, WA 6001 Australia

**Keywords:** Arbuscular mycorrhizal fungi, Ion balance, Chloroplast, Salt tolerance, Maize

## Abstract

**Background:**

Inoculation of arbuscular mycorrhizal (AM) fungi has the potential to alleviate salt stress in host plants through the mitigation of ionic imbalance. However, inoculation effects vary, and the underlying mechanisms remain unclear. Two maize genotypes (JD52, salt-tolerant with large root system, and FSY1, salt-sensitive with small root system) inoculated with or without AM fungus *Funneliformis mosseae* were grown in pots containing soil amended with 0 or 100 mM NaCl (incrementally added 32 days after sowing, DAS) in a greenhouse. Plants were assessed 59 DAS for plant growth, tissue Na^+^ and K^+^ contents, the expression of plant transporter genes responsible for Na^+^ and/or K^+^ uptake, translocation or compartmentation, and chloroplast ultrastructure alterations.

**Results:**

Under 100 mM NaCl, AM plants of both genotypes grew better with denser root systems than non-AM plants. Relative to non-AM plants, the accumulation of Na^+^ and K^+^ was decreased in AM plant shoots but increased in AM roots with a decrease in the shoot: root Na^+^ ratio particularly in FSY1, accompanied by differential regulation of ion transporter genes (i.e., *ZmSOS1*, *ZmHKT1*, and *ZmNHX*). This induced a relatively higher Na^+^ efflux (recirculating) rate than K^+^ in AM shoots while the converse outcoming (higher Na^+^ influx rate than K^+^) in AM roots. The higher K^+^: Na^+^ ratio in AM shoots contributed to the maintenance of structural and functional integrity of chloroplasts in mesophyll cells.

**Conclusion:**

AM symbiosis improved maize salt tolerance by accelerating Na^+^ shoot-to-root translocation rate and mediating Na^+^/K^+^ distribution between shoots and roots.

**Supplementary Information:**

The online version contains supplementary material available at 10.1186/s12870-021-03237-6.

## Background

Soil salinization and its associated land degradation is a growing environmental risk for crop production, affecting >800 million hectares of land globally [1], especially in the arid and semi-arid areas with limited rainfall and high evapotranspiration. Halophytes can adapt to salt stress and sustain the normal development. However, glycophytes such as maize (*Zea mays* L.) and many other crop species are vulnerable to high concentrations of salt, and hence whose productivity is severely declined in saline soil [2]. Thereby, investigating the potential strategies for improving maize plants tolerance and productivity under salt stress is both an urgency and a challenge.

In saline soils, Cl^−^ is considered the more toxic ion for some crop and woody species, such as legumes, citrus and grapevine [3]. For most crop species including maize, Na^+^ (rather than Cl^−^) was the primary ion causing the toxicity in relation to salinity [1]. Plant responses to salt stress involve restricted plant-available water induced by early-occurring osmotic stress, and reduced plant-growth rate that is accompanied by a Na^+^-specific component causing biochemical perturbations [1, 4, 5]. More harsh plant damage occurs in the latter phase of salt stress when excess cytosolic Na^+^ components displace K^+^, causing stomatal regulation disturbance [6], chloroplast deformation and malfunction, and reduced enzyme activation and protein biosynthesis [7]. It has been observed that when cultivated under salinity, crop plants, such as wheat, rice and maize, decrease in K^+^: Na^+^ ratios relative to the non-salinized counterparties [8–10], and structural components of chloroplast, such as membrane, grana and thylakoids, are seriously damaged at high salinity concurrent with interrupted metabolism in the mesophyll cell [11]. Consequently, preventing Na^+^ over-accumulation and maintenance of K^+^: Na^+^ balance (“homeostasis” as used in many publications) in the cytoplasm is crucial for salt-stressed plants. Long-distance Na^+^ translocation from roots to shoots occurs through the xylem and the main site of Na^+^ toxicity is in the shoots [12]. As such, strategies that accelerate Na^+^ removal from shoots while retaining K^+^ will contribute to salt tolerance [13–15]. Plasma membrane-localized Na^+^/H^+^ antiporter SOS1 is implicated in Na^+^ exclusion from the cytosol into the apoplast and the control of long-distance Na^+^ transport for xylem loading [16]; vacuolar sequestration of Na^+^ catalyzed by the tonoplast Na^+^/H^+^ antiporters (NHX) [17, 18] minimizes Na^+^ toxicity and simultaneously assists in osmotic adjustment [19]. They are the two major strategies for controlling cytosolic Na^+^ accumulation. Moreover, class I HKT transporters function in the xylem parenchyma to unload Na^+^ from the xylem stream [20, 21]. On the other hand, Na^+^ from photosynthetic tissues could be retrieved by the action of HKT1 transporters at phloem companion cells and this may result in significant Na^+^ recirculation through phloem. This transport process of Na^+^ is demonstrated in maize [22] and sweet pepper [23]. In the xylem parenchyma cell, the stelar K^+^ outward rectifier (SKOR) channel mediates K^+^ efflux for K^+^ release into the xylem [24]. Evidence showed that passive secretion of K^+^ into the xylem stream through SKOR channels can occur, contributing to (~50%) K^+^ translocation towards the shoot [25].

Intrinsic protective systems aside, plants can counter salinity stress by associating with beneficial soil microorganisms, such as arbuscular mycorrhizal (AM) fungi. Studies have shown that AM fungi facilitate the uptake of K^+^ but limit Na^+^ absorption and translocation to shoot tissues in plants exposed to salinity [26–28]. Mycorrhizal plants tend to have higher K^+^: Na^+^ ratios and lower shoot Na^+^ concentrations than non-mycorrhizal plants grown in saline soils, which prevent disturbances in cytosolic enzymatic processes and protein synthesis [29, 30]. This indicates that AM fungi help to ameliorate NaCl-induced ionic imbalance [29] and maintain the structural and functional integrity of cells and/or organelles [11, 31].

Little is known about how AM symbioses regulate the expression of plant genes encoding ion transporters involved in Na^+^/K^+^ balance, the process that underpins the mechanisms controlling plant tolerance to salinity stress. Ouziad et al. [32] showed that the transcript levels of two Na^+^/H^+^ antiporter genes (*LeNHX1* and *LeNHX2*) was not altered by AM colonization. However, Estrada et al. [33] reported that native AM fungi isolated from saline habitats regulated root *ZmAKT2*, *ZmSOS1* and *ZmSKOR* expression more than non-native AM fungi, as indicated by the higher K^+^: Na^+^ ratios in plants inoculated with native AM fungi. Porcel et al. [10] found that AM symbiosis improved salt tolerance in plants by decreasing the root-to-shoot distribution of Na^+^ through the regulation of *OsSOS1*, *OsNHX3*, *OsHKT1;5* and *OsHKT2;1*. Similarly, upregulated root expression of *RpSOS1*, *RPHKT1*, and *RpSKOR* in AM plants grown under salinity contributed to the K^+^: Na^+^ balance and lower shoot: root Na^+^ ratio, thus alleviating salt stress [34].

Root systems anchor the plant in the soil, and are actively implicated in the perception and transduction of the stress-induced signal that lead to morphological responses [35]. Accordingly, the development of root is plastic and can be modified to the stressful environments not only for improved scavenging for water and nutrients but also for diminished exposure to stress including salinity [36]. AM colonization has been shown to affect the root morphology in response to salinity. For example, an increase in root length and root mass of pepper [37], an increase in root diameter, root volume and root mass while a decrease in specific root length of maize [38]. However, the effect of AM fungi on the root morphology of maize grown under salinity is still rarely investigated.

The use of genotypes with contrasting root systems or salt tolerance may help to elucidate the possible role of AM fungi in alleviating salt tolerance in hosts. Thus, this study used two maize genotypes with contrasting root system size and salt tolerance selected from our previous experiments [9, 39]. The purposes of this study were to examine (1) alterations in root morphology, (2) the expression of key plant ion transporters involved in Na^+^/K^+^ balance, and (3) alterations in ultrastructure of chloroplasts in leaves of the two maize genotypes following AM inoculation under salt stress. The outcomes of this study would shed the light on the tolerance mechanisms of AM plants against salinity.

## Results

### Mycorrhizal colonization

**Fig. 1:**
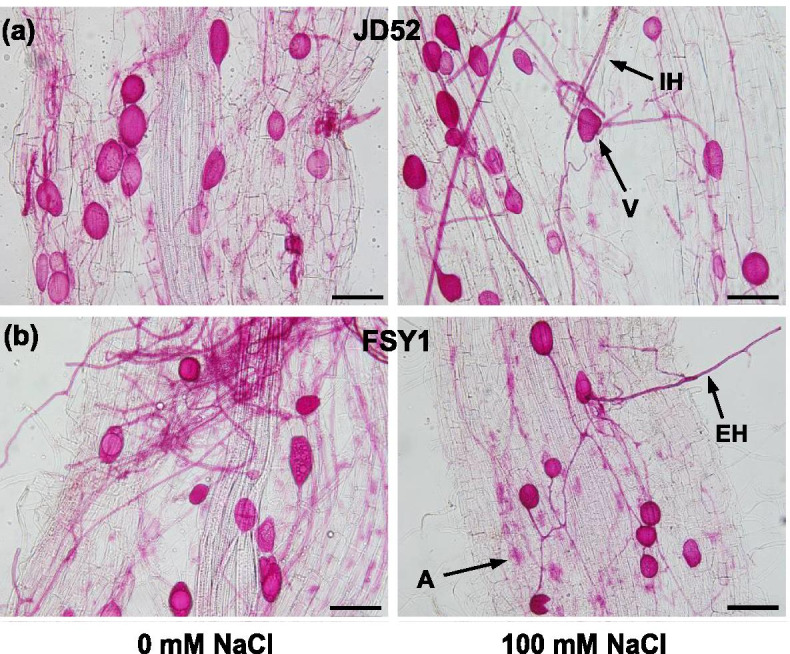
Microscopy root images of maize genotypes JD52 (**a**) and FSY1 (**b**) inoculated with arbuscular mycorrhizal fungus (*Funneliformis mosseae*) in 0 (left) and 100 mM NaCl (right) treatments assessed 59 days after sowing (DAS). Typical AM structures: A, arbuscule; IH, intraradical hyphae; EH, extraradical hyphae; V, vesicle. Bar = 100 μm

Both maize genotypes had high mycorrhizal colonization rates: 88 and 93% (without NaCl), and 85 and 91% (100 mM NaCl) in JD52 and FSY1, respectively (Table 1). No significant differences in mycorrhizal colonization rates were observed between 0 and 100 mM NaCl in either genotype. Typical microscopy structures of AM, such as vesicles, intraradical or extraradical hyphae, were observed in root samples of the inoculated plants (Fig. 1). Uninoculated plants did not develop mycorrhizal association.

**Table 1:** Mycorrhizal colonization rate and shoot and root traits of maize genotypes (JD52 and FSY1) inoculated with arbuscular mycorrhizal fungus (*Funneliformis mosseae*) (AM) or without inoculation (NM) in 0 and 100 mM NaCl treatments assessed 59 days after sowing (DAS)

	AM (%)	Shoot biomass (mg plant^−1^)	Root biomass (mg plant^−1^)	Total root length (cm)	Root diameter (mm)	Root surface area (cm^2^)	Root volume (cm^3^)	Root-shoot ratio	Specific root length (cmmg^−1^)	Root tissue density (mgcm^−3^)
**Genotype JD52**										
0 mM NaCl										
NM	0 b	2107 ± 70.58 b	971 ± 22.58 c	1348 ± 155.99 bc	1.35 ± 0.15 b	404 ± 50.56 b	9.97 ± 1.08 b	0.46 ± 0.01 a	1.39 ± 0.14 a	101 ± 8.93 b
AM	88 ± 1.52 a	3675 ± 101.89 a	1686 ± 108.23 a	1887 ± 117.02 a	1.96 ± 0.07 a	578 ± 39.28 a	13.1 ± 0.71 a	0.46 ± 0.03 a	1.27 ± 0.09 a	134 ± 10.02 a
100 mM NaCl										
NM	0 b	1849 ± 40.09 b	745 ± 51.99 d	1140 ± 71.19 c	1.00 ± 0.05 c	320 ± 12.86 b	8.67 ± 0.42 b	0.40 ± 0.03 a	1.37 ± 0.08 a	86.3 ± 5.76 b
AM	85 ± 2.10 a	3330 ± 150.28 a	1346 ± 11.67 b	1614 ± 35.92 ab	1.08 ± 0.02 c	376 ± 14.43 b	9.59 ± 0.55 b	0.41 ± 0.02 a	1.20 ± 0.03 a	125 ± 2.23 a
**Genotype FSY1**										
0 mM NaCl										
NM	0 b	1984 ± 145.32 bc	876 ± 94.82 c	1068 ± 13.33 c	1.07 ± 0.05 b	342 ± 19.07 bc	9.25 ± 0.87 ab	0.45 ± 0.05 bc	1.28 ± 0.14 a	95 ± 5.44 b
AM	93 ± 1.71 a	2635 ± 230.14 a	1606 ± 54.44 a	1750 ± 93.45 a	1.65 ± 0.12 a	537 ± 61.72 a	11.9 ± 1.73 a	0.62 ± 0.05 a	1.15 ± 0.05 a	121 ± 4.34 a
100 mM NaCl										
NM	0 b	1588 ± 153.70 c	590 ± 79.82 d	883 ± 91.05 c	0.90 ± 0.06 b	269 ± 44.17 c	6.25 ± 1.49 b	0.37 ± 0.03 c	1.23 ± 0.11 a	87.3 ± 6.95 b
AM	91 ± 1.63 a	2158 ± 156.01 ab	1171 ± 90.27 b	1427 ± 71.88 b	1.53 ± 0.21 a	404 ± 32.22 b	11.6 ± 0.87 a	0.54 ± 0.04 ab	1.13 ± 0.04 a	112 ± 3.68 a

Notes: AM (%) = root length colonization rate (%). Data are means ± SE (*n* = 5), for each genotype, data of traits followed by different letters are significantly different (*P* ≤ 0.05)

### Plant shoot and root traits

The 100 mM NaCl treatment reduced the biomass production in maize, more so in roots than shoots, such that root: shoot ratio slid (Table 1; Table 2). Salt stress reduced shoot and root growth more in the salt-sensitive genotype FSY1 than in the salt-tolerant genotype JD52. Plants with AM inoculation had higher biomass than non-AM plants; the inoculation effect was greater for shoot growth in JD52, and for root growth in FSY1 (Table 1; Fig. S1). In non-AM plants, the 100 mM NaCl treatment reduced shoot dry weight by 12 and 20% in JD52 and FSY1, respectively, relative to the 0 mM NaCl treatment (control). The corresponding reductions in the AM plants were 9 and 18%. Compared to the non-AM treatment, AM inoculation significantly increased shoot dry weight by 74% (JD52) and 33% (FSY1) at 0 mM NaCl, and 80% (JD52) and 36% (FSY1) at 100 mM NaCl.

**Table 2:** Three-way analysis of variance (ANOVA)

Traits measured	G	S	AM	G × S	G × AM	S × AM	G × S × AM
AM colonization	***	*	***	n.s.	***	*	n.s.
Shoot biomass	***	***	***	n.s.	***	n.s.	n.s.
Root biomass	*	***	***	n.s.	n.s.	n.s.	n.s.
Total root length	**	***	***	n.s.	n.s.	n.s.	n.s.
Root diameter	n.s.	***	***	**	n.s.	n.s.	n.s.
Root surface area	n.s.	***	***	n.s.	n.s.	n.s.	n.s.
Root volume	n.s.	**	***	n.s.	n.s.	n.s.	n.s.
Root-shoot ratio	*	**	***	n.s.	***	n.s.	n.s.
Specific root length	n.s.	n.s.	n.s.	n.s.	n.s.	n.s.	n.s.
Root tissue density	n.s.	*	***	n.s.	n.s.	n.s.	n.s.
Shoot Na^+^ content	n.s.	***	*	n.s.	n.s.	n.s.	n.s.
Root Na^+^ content	n.s.	***	**	n.s.	n.s.	**	n.s.
Shoot K^+^ content	***	**	n.s.	n.s.	n.s.	*	n.s.
Root K^+^ content	n.s.	n.s.	n.s.	n.s.	n.s.	n.s.	n.s.
Shoot K^+^:Na^+^ ratio	***	***	n.s.	**	n.s.	n.s.	n.s.
Root K^+^:Na^+^ ratio	n.s.	**	n.s.	n.s.	n.s.	n.s.	n.s.
Shoot: root Na^+^ ratio	n.s.	**	***	n.s.	n.s.	n.s.	n.s.
*SOS1* shoot	***	**	**	n.s.	n.s.	n.s.	n.s.
*SOS1* root	n.s.	**	***	n.s.	n.s.	n.s.	n.s.
*HKT1* shoot	*	*	n.s.	*	*	n.s.	n.s.
*HKT1* root	***	**	n.s.	**	n.s.	n.s.	n.s.
*NHX* shoot	***	***	*	n.s.	n.s.	*	*
*NHX* root	***	***	n.s.	***	***	n.s.	***
*SKOR* shoot	***	***	n.s.	***	n.s.	n.s.	n.s.
*SKOR* root	n.s.	***	n.s.	n.s.	n.s.	n.s.	n.s.

The sources of variation are genotype (G), salt treatment (S), AM inoculation (AM) and the interactions (G × S, G × AM, S × AM, G × S × AM)

*n.s.* not significant

*P ≤ 0.05

**P ≤ 0.01

***P ≤ 0.001

Relative to the control, the 100 mM NaCl treatment significantly reduced root dry weight in the non-AM plants by 23% (JD52) and 33% (FSY1). For the AM plants, the 100 mM NaCl treatment decreased root dry weight by 20% (JD52) and 27% (FSY1), relative to the control. Compared to the non-AM treatment, root dry weight increased significantly with AM inoculation, by 74% (JD52) and 83% (FSY1) at 0 mM NaCl, and 81% (JD52) and 98% (FSY1) at 100 mM NaCl.

In non-AM plants, total root length declined to a similar level in both genotypes—15% (JD52) and 17% (FSY1) at 100 mM NaCl, relative to the control. The corresponding reductions in the AM plants were 14 and 18%. Compared to the non-AM treatment, root length significantly increased with AM inoculation, by 40% (JD52) and 64% (FSY1) at 0 mM NaCl, and 41% (JD52) and 62% (FSY1) at 100 mM NaCl (Tables 1 and 2).

The 100 mM NaCl treatment also negatively affected root diameter, root surface area, root volume, specific root length and root tissue density (Tables 1 and 2). The AM plants had significantly higher values for root diameter, root surface area, and root volume than the non-AM plants, except in JD52 at 100 mM NaCl, where the differences were not significant. AM inoculation also significantly increased root tissue density in both genotypes, but only slightly decreased specific root length.

### Accumulation of Na^+^ and K^+^ in shoots and roots

**Fig. 2:**
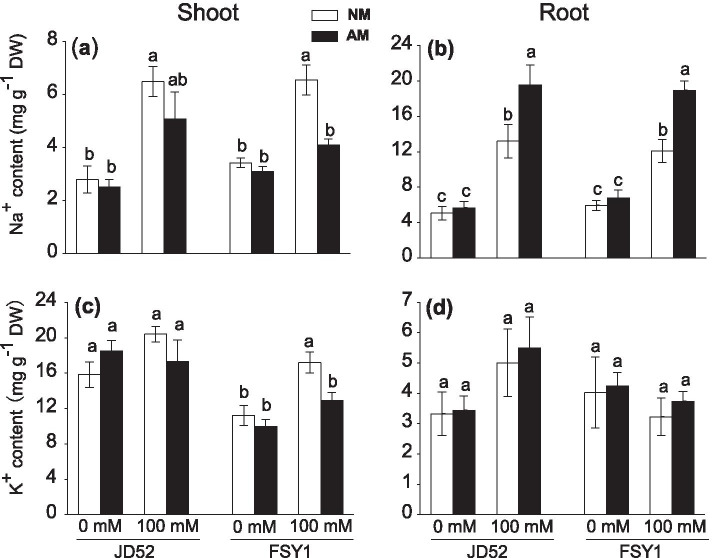
K^+^ and Na^+^ contents in shoots (**a, c**) and roots (**b, d**) of maize genotypes JD52 and FSY1 inoculated with arbuscular mycorrhizal fungus (*Funneliformis mosseae*) (AM) or without inoculation (NM) in 0 and 100 mM NaCl treatments assessed at 59 DAS. Data are means ± SE (*n* = 5), in each graph, data of traits were compared within each genotype, bars with different letters are significantly different (*P ≤* 0.05)

The accumulation of Na^+^ in shoots and roots increased notably when maize plants were treated with 100 mM NaCl (Fig. 2a, b; Table 2). Shoot and root Na^+^ contents did not significantly differ between non-AM and AM plants in both genotypes at 0 mM NaCl. However, at 100 mM NaCl, AM inoculation decreased shoot Na^+^ content in FSY1 (the decrease was not significant in JD52, *P* > 0.05) (Fig. 2a), whereas opposite results were found for root Na^+^ accumulation (Fig. 2b).

The 100 mM NaCl treatment did not affect shoot K^+^ accumulation in both genotypes regardless of AM inoculation, except for non-AM FSY1 plants, which accumulated higher K^+^ level than the non-salt control (Fig. 2c; Table 2). In both genotypes, non-AM and AM plants had similar shoot K^+^ contents at 0 mM NaCl, but at 100 mM NaCl, AM inoculation decreased shoot K^+^ content in FSY1 (the decrease in JD52 was moderate, *P* > 0.05). No significant differences in root K^+^ accumulation were observed in either genotype, regardless of salt or inoculation treatments (Fig. 2d).

### Tissue K^+^: Na^+^ ratio, and shoot: root Na^+^ ratio

**Fig. 3:**
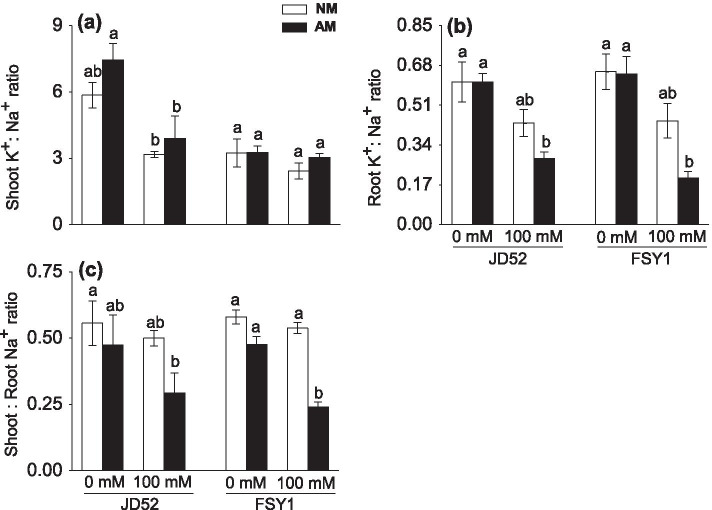
K^+^: Na^+^ ratios in shoots (**a**) and roots (**b**), and shoot: root Na^+^ ratio (**c**) of maize genotypes JD52 and FSY1 inoculated with arbuscular mycorrhizal fungus (*Funneliformis mosseae*) (AM) or without inoculation (NM) in 0 and 100 mM NaCl treatments assessed at 59 DAS. Data are means ± SE (*n* = 5), in each graph, data of traits were compared within each genotype, bars with different letters are significantly different (*P* ≤ 0.05)

The shoot and root K^+^: Na^+^ ratios were negatively affected by NaCl application in both genotypes with or without AM inoculation (Fig. 3a, b; Table 2). At 0 or 100 mM NaCl, AM inoculation moderately enhanced the shoot K^+^: Na^+^ ratio but decreased the root K^+^: Na^+^ ratio in both genotypes. The salt tolerant genotype JD52 had higher shoot K^+^: Na^+^ ratios than the sensitive genotype FSY1, regardless of salt or inoculation treatments.

As an indication of Na^+^ translocation from roots to shoots, the shoot: root Na^+^ ratio tended to decline upon exposure to salinity in both genotypes with or without AM inoculation (Fig. 3c; Table 2). At 100 mM NaCl, AM inoculation significantly decreased the shoot: root Na^+^ ratio in FSY1 (the decrease was moderate in JD52, *P* > 0.05).

### Expression of *ZmSOS1*, *ZmHKT1*, *ZmNHX* and *ZmSKOR*

**Fig. 4:**
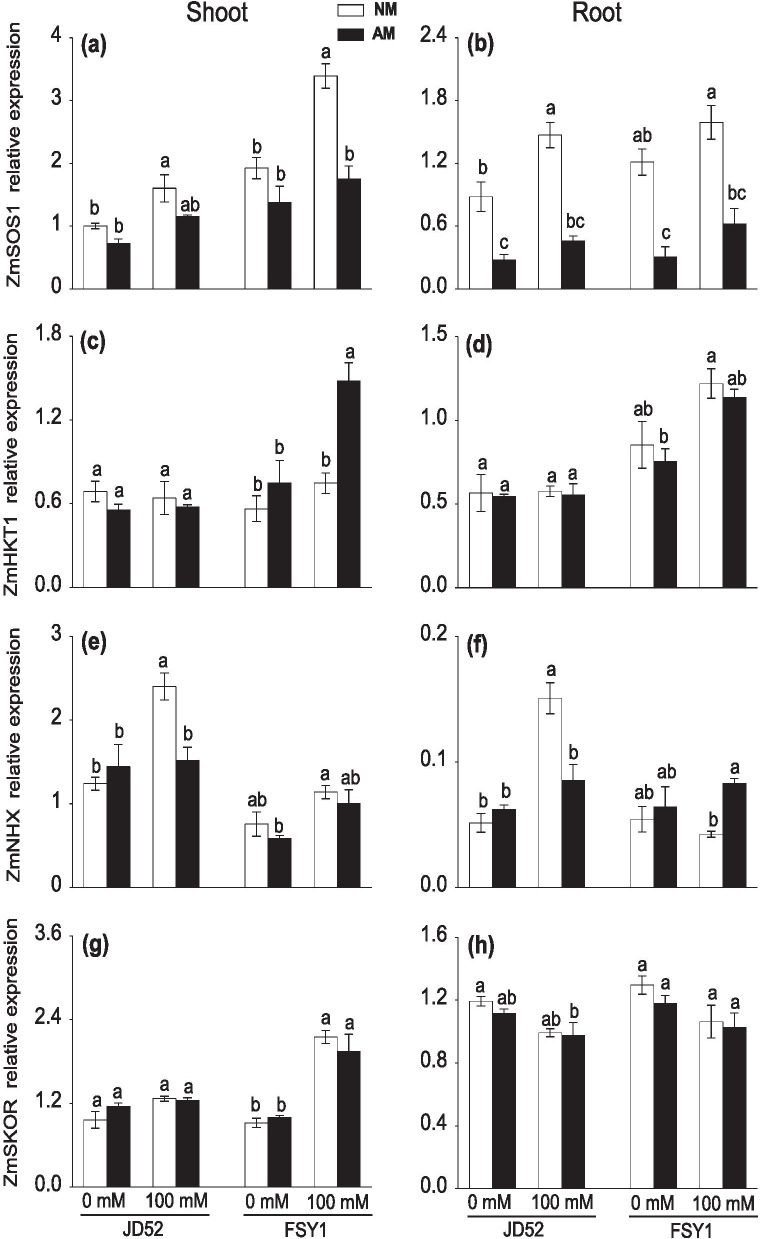
Expression of ion transporter genes (*ZmSOS1*, *ZmHKT1*, *ZmNHX*, and *ZmSKOR*) in shoots (**a, c, e, g**) and roots (**b, d, f, h**) of maize genotype JD52 and FSY1 inoculated with arbuscular mycorrhizal fungus (*Funneliformis mosseae*) (AM) or without inoculation (NM) in 0 and 100 mM NaCl treatments assessed at 59 DAS. Data are means ± SE (*n* = 3), in each graph, data of traits were compared within each genotype, bars with different letters are significantly different (*P ≤* 0.05)

Relative to the non-salt control, the application of 100 mM NaCl significantly increased the *ZmSOS1* expression in shoots and roots of both genotypes without AM inoculation, except in roots of FSY1, where the expression of this gene changed slightly compared to the non-salt control. The 100 mM NaCl application did not affect the *ZmSOS1*expression in tissues of either genotype with AM inoculation. However, AM inoculation has downregulated this gene expression in shoots of FSY1 and roots of both genotypes at 100 mM NaCl (Fig. 4a, b; Table 2).

In shoots, salt application or AM inoculation had little effect on the expression of *ZmHKT1* in JD52. However, the 100 mM NaCl treatment increased *ZmHKT1* expression in FSY1 with AM inoculation. AM inoculation upregulated *ZmHKT1* expression in shoots of FSY1 at 100 mM NaCl, with an almost two-fold increase relative to the corresponding non-AM plants (Fig. 4c). Salt application did not affect *ZmHKT1* expression in roots with or without AM inoculation. AM inoculation slightly decreased the expression of this gene in roots in either genotype at 0 or 100 mM NaCl (Fig. 4d; Table 2).

The 100 mM NaCl treatment enhanced *ZmNHX* expression in shoots of JD52 without AM inoculation. The expression of *ZmNHX* was not affected by AM inoculation except in JD52 at 100 mM NaCl, where AM plants had significantly lower expression than non-AM plants (Fig. 4e). The 100 mM NaCl treatment little affected *ZmNHX* expression in roots with or without AM inoculation, except for non-AM JD52 plants, which had significantly higher expression than the 0 mM NaCl control. AM inoculation had little effect on the expression of *ZmNHX* at 0 mM NaCl, but significantly inhibited *ZmNHX* expression in JD52 while upregulated it in FSY1 at 100 mM NaCl (Fig. 4f).

In the 100 mM NaCl treatment, the expression of *ZmSKOR* was increased in shoots of FSY1 with or without AM inoculation, relative to the non-salt control (Fig. 4g). However, salt application slightly decreased this gene expression in roots with or without AM inoculation (Fig. 4h). The *ZmSKOR* expression was not affected by AM inoculation in either genotypes at 0 and 100 mM NaCl, but its overall expression was more pronounced in FSY1 than JD52.

### Ultrastructures of chloroplast

**Fig. 5:**
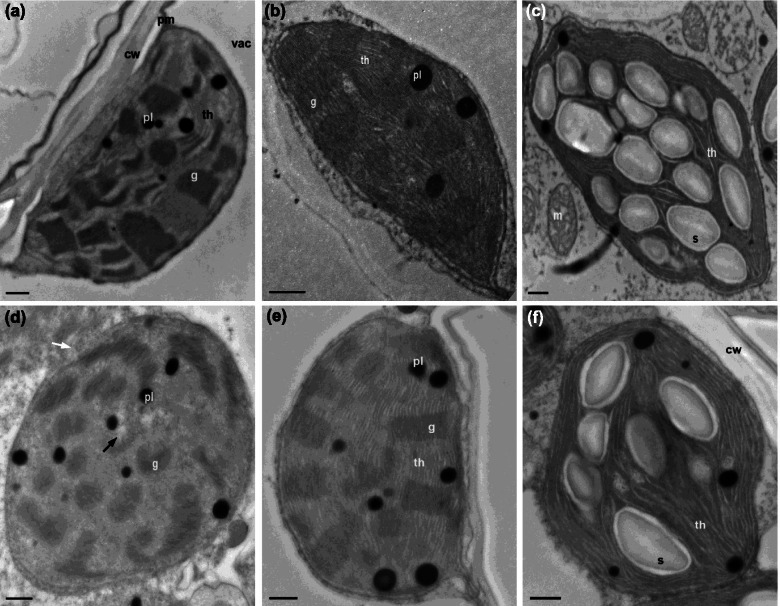
Transmission electron images of chloroplasts in mesophyll cells in maize genotype JD52. **a** non-AM (NM) control plants: somewhat-inflated chloroplast with slightly disaggregated and swollen grana and thylakoids. **b,c** AM control plants: elongated chloroplasts with compactly stacked grana lamella and thylakoids, enveloped by a well-defined membrane. Note fewer plastoglobules, and often abundant starch grains within chloroplasts. **d** NM plants exposed to 100 mM NaCl: oval-shaped chloroplast with a degree of membrane degradation (white arrow), loose and swollen granal lamella, disorganized and partially dissolved thylakoids (black arrow), more plastoglobules but absence of starch formation. **e, f** AM plants exposed to 100 mM NaCl: structure of grana stacks and thylakoids remain in contact, number of plastoglobules decreased but size increased, some starch grains still formed occasionally. cw, cell wall; pm, plama membrane; vac, vacuole; th, thylakoid; g, grana; pl, plastoglobule; s, starch grain; m, mitochondria. Bar = 0.5 μm

**Fig. 6:**
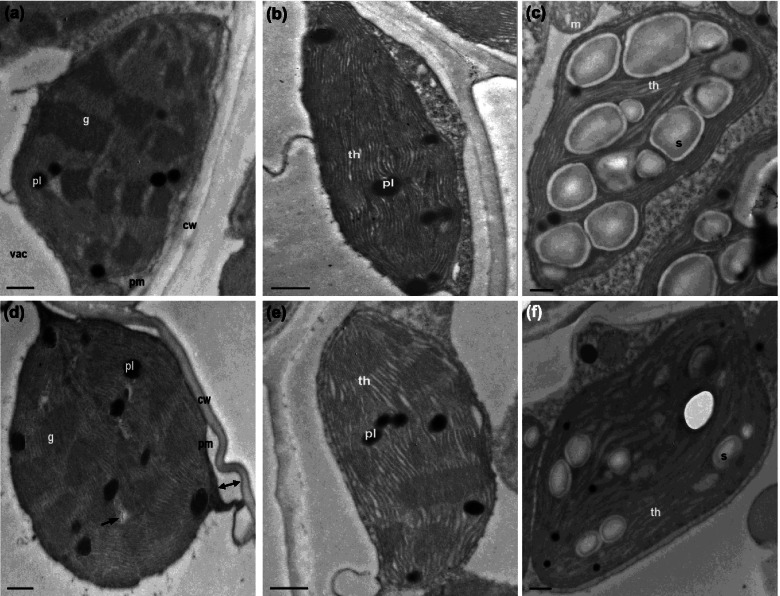
Transmission electron images of chloroplasts in mesophyll cells in maize genotype FSY1. **a** NM control plants: slightly inflated chloroplast with some swollen grana lamella. **b, c** AM control plants: elongated chloroplasts with thylakoids compactly stacked in stroma and a complete membrane. Note fewer plastoglobules, and mostly numerous starch grains within chloroplasts. **d** NM plants exposed to 100 mM NaCl: sphere-shaped chloroplast with corrugated membrane, most granal stacks have disintegrated, thylakoids cavitated at certain places (black arrow), plastoglobules increased but starch grains absent, and plasmolysis initiated (double arrow). **e, f** AM plants exposed to 100 mM NaCl: grana stacks and thylakoids remain in contact, with fewer plastoglobules and sometimes starch grain formation. cw, cell wall; pm, plama membrane; vac, vacuole; th, thylakoid; g, grana; pl, plastoglobule; s, starch grain; m, mitochondria. Bar = 0.5 μm

Without NaCl application, the mesophyll cells in non-AM maize plants of both genotypes contained slightly inflated chloroplasts. Several grana and thylakoids also showed signs of swelling and disaggregating (Figs. 5 and 6a). The corresponding AM plants had prominent, elongated chloroplasts with closely arranged thylakoids and well-compartmentalized grana stacked in stroma enveloped by complete double-layered membranes. In particular, they had a few plastoglobules and often filled with starch grains (Figs. 5 and 6b, c). When exposed to 100 mM NaCl, the chloroplast untrastructures in non-AM plants were characterized by a paucity of degraded membrane, loose and swollen granal lamella, partially dissolved or even cavitated thylakoids, increased accumulation of plastoglobules and persisted absence of starch reserves (Fig. 5d). However, the damaging effects of salt stress on the chloroplast were more apparent in FSY1, which was spherically shaped with membrane corrugated and most granal stacks disintegrated, and initiated plasmolysis was observed as well (Fig. 6d). The corresponding chloroplasts in AM plants at 100 mM NaCl showed slight inflation with some swollen thylakoids, nevertheless, there was a strong formation of starch grains, particularly in JD52, and a decrease in plastoglobules, and intact granal stacks and compacted arrangement of thylakoids were maintained (Figs. 5 and 6e, f).

## Discussion

Plant biomass is an integrative trait that reflects plant performance grown under stressful conditions and the inoculation efficiency of AM fungi [40]. In the present study, both maize genotypes inoculated with AM fungus *F*. *mosseae* grew better under salt stress with higher plant biomass than uninoculated plants, suggesting that this AM fungal isolate alleviates salt stress. Salt stress did not significantly affect root colonization in both maize genotypes in this study, although it is generally accepted that salinity has negative effects on AM fungal colonization ability by inhibiting spore germination, sporulation and hyphal growth [41, 42]. Salt stress decreased the values of various root traits (root dry weight, total root length, etc.) in the present study, however, the AM plants had larger root system (root dry weight) than their non-AM counterparts. Although greater inoculation effect on shoot dry weight in JD52, the positive effect of AM fungal inoculation on root traits such as root biomass was more pronounced in FSY1 relative to JD52, which could be attributed to higher rate of root colonization by the fungus that can demand excess photosynthates from the shoot tissues in AM plants [43] of FSY1, especially under salt stress. Sheng et al. [38] reported that mycorrhizal maize plants had a coarser root system than their non-AM counterparts, and such change in root morphology was associated with the improved salt tolerance. Our results demonstrated that relative to non-AM plants, AM plants generally had higher root tissue density, suggesting that AM inoculation induced a shift towards a denser root system, and this may confer the tolerance of maize plants to Na^+^ toxicity and presumably correlating with the Na^+^ (K^+^) uptake and translocation, and the lower root K^+^: Na^+^ ratio and shoot: root Na^+^ ratio (Fig. 3b, c).

Na^+^ ions tend to build up in tissues of plants grown under salinity, which compete with K^+^ for metabolic processes requiring K^+^ [44]—salt tolerance in these plants will depend on the uptake, translocation and redistribution of Na^+^ within the plant. Previous studies revealed that AM plants can increase K^+^ uptake while decreasing Na^+^ accumulation in the cytoplasm under saline conditions as compared to non-mycorrhizal plants [30, 45]. However, we found that in AM plants grown under salt stress, both K^+^ and Na^+^ ions decreased in shoots but increased in roots, and with a concomitant increase in shoot K^+^: Na^+^, relative to non-AM plants. This somewhat contradictory result could be attributed to different molecular processes involved in salt-stressed plants. In addition, under salt stress, AM plants had consistently lower shoot: root Na^+^ ratios, particularly in FSY1, which suggests that restricting delivery of Na^+^ from roots to shoots and (or) recirculating Na^+^ to roots by long-distance transport serves as an essential strategy to prevent toxic Na^+^ levels in photosynthetic tissues. Similar findings have been reported for other plant species including wheat, rice, fenugreek and black locust [10, 29, 34]. It has also been proposed that Na^+^ could be stored inside root cell vacuoles, vesicles or intraradical fungal hyphae in AM-inoculated plants to reduce Na^+^ allocation to shoots [46]. However, the molecular basis for the possible ion transporter regulation mediated by AM fungi that controls the Na^+^ and K^+^ transport processes, needs further examination.

After an initial increase in Na^+^ in salinized plant tissues, cation transporters (SOS1, HKT and NHXs) function to restrict and translocate Na^+^ and indirectly control intracellular K^+^ balance [28]. At 100 mM NaCl, AM inoculation downregulated shoot *ZmSOS1* expression in FSY1 and root *ZmSOS1* expression (Fig. 4a, b), and upregulated shoot *ZmHKT1* expression in FSY1 (Fig. 4c). These results suggest that *ZmSOS1* mediates xylem Na^+^ loading [47], and downregulaion of *ZmSOS1* in AM roots reduces Na^+^ loading in the xylem, thereby increasing root Na^+^ accumulation. Meanwhile, upregulation of *ZmHKT1* in AM shoots of FSY1, in possible combination of the inhibited *ZmSOS1* activity, increased Na^+^ unloading from the xylem and recirculation from leaf tissues to roots. This is consistent with the results of Porcel et al. [10] who reported a similar expression profile for *OsSOS1* and *OsHKT1* in rice roots colonized by *Claroideoglomus etunicatum* and exposed to high NaCl concentrations. However, other studies showed that AM colonization increased *SOS1* and *HKT1* expressions in roots and this correlated with a decline in root Na^+^ accumulation [33, 34]. These inconsistencies might be a consequence of different methods of salt application, intensity and duration of salinity stress used in the experiments, for example, in the experiment of Chen et al. [34], the same amount dose of salt (100 or 200 mM NaCl) was continuously added every day for one week and then the plants were maintained under such conditions for additional three weeks. Indeed, the transport functions of SOS1 and HKT1 are coordinated to achieve Na^+^ partitioning and balance within plant tissues [47]. Zhu et al. demonstrated that *HKT1* genes affected the activity and transcript levels of SOS1 Na^+^/H^+^ exchanger in both cortical and stelar tissues in wheat [48].

The compartmentation of excessive Na^+^ into vacuoles, mediated by tonoplast-associated Na^+^/H^+^ antiporters (NHX), is a typical strategy for avoiding cytosolic Na^+^ accumulation in relation to salt tolerance [49]. Increased transcription of *ZmNHX* in plant roots reduces Na^+^ transport to the root xylem vessels under salt stress and thus excluding Na^+^ from shoots [27]. After 100 mM NaCl treatment, shoot *ZmNHX* expression was significantly upregulated in JD52 but changed little in FSY1 without AM inoculation (Fig. 4e), which is coherent with the hypothesis that salt tolerant genotypes have significantly higher transcript levels of *NHX* in shoots than salt sensitive genotypes under salinity [27, 50]. It is likely that the downregulation of *ZmNHX* by AM inoculation in roots of JD52 under salt stress (Fig. 4f) may be associated with its tolerance to the Na^+^ toxicity and therefore declined inclusion of Na^+^ in the root vacuoles. Conversely, under salt stress, upregulation of *ZmNHX* in the roots of FSY1 by AM inoculation increased Na^+^ sequestration into root vacuoles and thus limited Na^+^ transport from roots to shoots; when combined with *ZmSOS1* and *ZmHKT1*, this may have contributed to the significantly lower shoot Na^+^ content and shoot: root Na^+^ ratio. Overall, AM inoculation exerted a more evident effect on the expression of these Na^+^ transporters in shoots than in roots, implicating that AM associations preferentially protect photosynthetic tissues from the Na^+^ toxicity over root tissues, and confirming that a critical feature of salinity tolerance is directly associated with the effective removal of Na^+^ from leaf blade in glycophytes [27]. In this respect, the AM inoculation was more effective in ameliorating Na^+^/K^+^ imbalance in salt-sensitive genotype FSY1 than JD52 in the present study.

The optimal cytosolic K^+^: Na^+^ ratio can be stabilized by either preventing Na^+^ accumulation or K^+^ loss from the cell; the retention of K^+^, specifically in roots, is central to salt tolerance [51, 52]. Under saline conditions, depolarization of the plasma membrane caused by Na^+^ influx favors the release of K^+^ into the xylem via depolarization-activated outward-rectifying K^+^ channels [53]. The accumulated evidence indicated that *SKOR* is upregulated in AM roots exposed to salinity and accounting for the increased K^+^ accumulation in shoots and improved K^+^: Na^+^ ratio [33, 34]. In contrast, we found that AM inoculation moderately inhibited the expression of *ZmSKOR* gene in shoot and root tissues of both genotypes under salt stress (Fig. 4g, h). Downregulation of *ZmSKOR* retarded the rate of K^+^ loading into the xylem stream and, eventually diminished K^+^ translocation to the shoot and facilitating root K^+^ retention. Moreover, it appears that relative to non-AM shoots, AM shoots had a relatively higher Na^+^ efflux (recirculating) rate than that of K^+^, which correlated with the improved K^+^: Na^+^ ratio, while the opposite was observed in roots.

The cytosolic K^+^: Na^+^ ratio, particularly in leaves, is a crucial trait used to characterize salt tolerance. AM plants tend to accumulate less Na^+^ and more K^+^ to achieve an adequate K^+^: Na^+^ ratio within the cytoplasm [30, 45]. Relative to the salinized non-AM counterparts, Na^+^ translocation to shoots declined in AM plants (Figs. 2a and 3c) and, despite the decline in shoot K^+^ content, particularly in FSY1 (Fig. 2c), both genotypes maintained adequate K^+^: Na^+^ ratios (Fig. 3a), thus contributing to ion balance in cell cytoplasm and better growth. Consequently, we concluded that salinity tolerance is not only related to the capacity to remove Na^+^ ions from shoots, but also to higher rates of Na^+^-translocation from shoots to roots than K^+^. These findings reconciled with the statement of Maathuis [28] that regulation of the rate of Na^+^ delivery to the shoot over time is crucial to plant salt tolerance.

Cell organelle chloroplasts are most sensitive to salt stress [54]. The present study revealed that exposure of non-AM maize plants to salt stress resulted in ultrastructural alterations in chloroplasts, particularly in FSY1 (Figs. 5 and 6d). However, some slightly disaggregated and swollen grana and thylakoids observed in chloroplasts of non-AM plants without NaCl may be attributable to nutrient deficiency or imbalance in shoots to a small extent (Fig. 3a, c), under the controlled nutrient conditions favorable for AM fungal growth. Salt-induced osmotic stress can cause thylakoid swelling and membrane damage [55]. With time, accumulated ion stress due to unfavorable shoot K^+^: Na^+^ ratio (Fig. 3a) leads to the thylakoid deformation and grana destacking [56]. In addition, the observed lack of starch grains suggests the assimilate metabolism is interrupted in chloroplasts [57]. It is likely that relative to non-AM plants, the reduced Na^+^ load in mesophyll cells helped to avoid Na^+^ toxicity and maintain the structural integrity and normal function of chloroplasts in AM plants. Also, the decreased number of plastoglobules in AM plants observed in our study might indicate the mitigated oxidative stress induced by NaCl, as observed by [31].

## Conclusions

The AM fungus *F. mosseae* improved the salt tolerance of maize by increasing tissue density, extruding Na^+^ from leaves, partitioning Na^+^ in plant organs, maintaining K^+^: Na^+^ balance, and preserving the structural integrity of organelles and their function. Differential regulation of cation transporter genes by AM fungi in both maize genotypes had a similar effect on shoot and root Na^+^ (K^+^) uptake, accumulation and the subsequent K^+^: Na^+^ and shoot: root Na^+^ ratios. Under salt stress, AM symbiosis better sustained shoot growth in the salt-tolerant genotype JD52 but root growth in the salt-sensitive genotype FSY1, which was directly related to a higher rate of Na^+^ recirculation from shoots to roots, relative to K^+^ and higher sink strength in FSY1. To verify the protective efficacy and mechanisms of AM symbiosis, the performance of salt tolerant (JD52) and sensitive (FSY1) maize genotypes in association with AM fungi warrants further study under salt-affected agricultural fields. Also, future studies are essential to validate these findings involving multiple plant genotypes and/or AM fungal isolates in saline soils.

## Methods

### Plant, soil and AM inoculum

Two maize (*Zea mays* L.) genotypes, salt-tolerant Jindan52 (JD52) with large root system size (in terms of total root length and root dry mass), and salt-sensitive Fushengyuan1 (FSY1) with small root system size, were selected from our recent root phenotyping study involving 174 maize genotypes [39], and a follow-up experiment characterizing plant response to salinity (with 100 mM NaCl as a threshold level) in 20 selected genotypes [9] using an established semi-hydroponic phenotyping platform.

A mixture of filed soil (<2.0 mm) and fine sand (<2.0 mm) (1:1, v/v) was used as the growth substrate. The mixed soil had a pH of 6.9 (soil: water, 1:2.5, w/v) and contained the following main nutrients (mg kg^−1^): N (36.4), P (16.6, NaHCO_3_-extractable), K (171) and organic matter (7200). The soil was autoclaved (0.11 MPa, 121 °C, 2 h) and shortly air-dried before filling in the non-draining plastic pots (200 mm in diameter, 160 mm high) with 2.68 kg soil per pot. The soil moisture content was 19.8% and the water content (w/w) at field capacity (i.e. pot capacity when fully drained) was 27.6%.

The AM fungus *Funneliformis mosseae* (BGC NM02A) was kindly provided by Institute of Mycorrhizal Biotechnology, Qingdao Agricultural University, Qingdao, China. The inoculum was propagated in pot (30 cm in diameter, 35 cm high) cultures of *Trifolium repens* L. and comprised a mixture of sand, spores (approximately 14 spores per gram), mycelia, and infected root fragments.

### Experimental design, planting and maintenance

A completely randomized design with two maize genotypes, two salinity levels (0 and 100 mM NaCl) and two inoculation treatments (non-AM and AM inoculation) was used in this pot experiment. The 100 mM NaCl was selected for this study, since maize genotypes showed higher sensitiveness to 100 mM NaCl than to other salinity levels, and the selected two genotypes (i.e., JD52 and FSY1) were ranked and assessed for salt tolerance based on the alterations in morphological and physiological traits under 100 mM NaCl compared to the non-saline control [9]. There were eight replicates for each treatment, totaling 64 pots (two plants per pot).

Similar-sized seeds of each maize genotype were surface sterilized in 10% H_2_O_2_ for 10 min, rinsed thoroughly with sterile water, and pre-germinated on sterilized moist filter paper for 2 days in darkness at 28 °C. Five uniform germinated seeds were sowed in each pot on 5 September 2018. For the AM treatment, each pot was supplied with 50 g of inoculum (containing around 700 infective propagules) just below the pre-germinated maize seeds at sowing, while the non-AM treatment received the same amount of autoclaved inoculum and the filtrate (<20 μm) of the AM inoculum to reintroduce a native microbial population free of AM propagules. Fourteen days after sowing (DAS), the seedlings were thinned to two per pot.

The experiment was carried out in a greenhouse, with natural light, average temperatures of 24/15 °C, and a relative humidity of 50–75%. Nutrient solution (50 ml) was supplied weekly containing (in μM): KH_2_PO_4_ (1000), KNO_3_ (5000), Ca(NO_3_)_2_ (7200), MgSO_4_ (4100), H_3_BO_3_ (46.3), MnCl_2_ (11.2), ZnSO_4_ (0.7), CuSO_4_ (0.32), H_2_MoO_4_ (0.1) and FeEDTA (20). All pots were randomized weekly to minimize positional effects.

The salt treatment (100 mM NaCl) commenced 32 DAS to allow plant and symbiosis establishment. To avoid osmotic shock, the 100 mM NaCl was added to pots in four equal doses on alternate days. Each dose contained 25 mM NaCl (0.32 g kg^−1^ soil), which was applied in 50 ml of de-ionized water to each pot and immediately a sufficient water volume added to wet the soil to 80% field capacity. The non-salt control pots were watered to 80% field capacity with an equivalent water volume. Pots were weighed daily and watered to maintain 80 ± 5% field capacity for the duration of the experiment. After four doses were added, the electrical conductivities in the soil were 0.2 and 8.2 dS m^−1^ for the 0 and 100 mM NaCl treatments, respectively.

### Plant harvesting and morphology assessments

Plants were assessed at 59 DAS. At which time, the shoots were cut at ground level and roots were gently washed free of soil (five replicate plants per treatment, two plants of the same pot were averaged as one replicate). A small fraction of roots from each pot were kept in 70% ethanol for mycorrhizal evaluation (see below for details). Shoots were oven-dried at 75 °C to constant weight to determine shoot dry weight (mg plant^−1^). Root samples were scanned with a flatbed scanner (Epson Perfection V800, USA) in grayscale at 300 dpi; root images were analyzed using WinRHIZO Pro (2009b, Regent Instruments, Montreal, QC, Canada) to generate morphology values (i.e., root length, average root diameter, root surface area and root volume). After scanning, root dry weight (mg plant^−1^) was measured after oven-drying as above. Specific root length was calculated as total root length/root dry weight (root length per unit mass). The root dry weight and root volume were used to calculate root tissue density (root mass per volume). Root to shoot ratio was calculated from root dry weight and shoot dry weight.

### Mycorrhizal colonization

A fraction of fresh roots were cleared with 5% KOH (90 °C, 20 min), acidified in 2% HCl (5 min, room temperature) and then stained in 0.01% acid fuchsin (overnight, room temperature) according to Kormanik et al. [58] and Liu and Chen [59]. Mycorrhizal colonization was measured using the gridline intersect method [60], in which 100 × 1 cm long root segments were mounted on glass slides and observed under a microscope (BX53 OLYMPUS, Japan). The percentage of mycorrhizal colonization (%) was calculated as the proportion of root fragments colonized by hyphae, arbuscules or vesicles.

### Tissue Na^+^ and K^+^

Oven-dried leaves and roots (100 mg) were separately ground into fine powder and digested in 5 ml H_2_SO_4_-H_2_O_2_, and then heated to 365 °C for extraction of Na^+^ and K^+^ ions. After cooling, samples of the digests were diluted to determine K^+^ and Na^+^ contents (mg g^−1^ dry weight) with a flame photometer (Flame Photometer 410, Sherwood, UK) [9]. K^+^: Na^+^ ratios and shoot: root Na^+^ ratios were calculated for each plant. There were five replicate plants per treatment.

### RNA extraction and cDNA synthesis

Fresh shoot and root samples (three plants per treatment) were frozen in liquid nitrogen and immediately stored at −80 °C until RNA isolation. Total RNA was extracted with the MiniBEST plant RNA extraction kit (Takara Bio, Dalian, China) and subjected to DNase treatment according to the manufacturer’s instructions. Complementary DNA (cDNA) was primed by random hexamer using 500 ng of DNase-treated RNA with the PrimeScript™ RT Mater Mix (Takara Bio, Dalian, China).

Primer sequences (ZmSOS1-F, ZmSOS1-R, ZmHKT1-F, ZmHKT1-R, ZmNHX-F, ZmNHX-R, ZmSKOR-F and ZmSKOR-R) used in the qRT-PCR are listed in Supplementary Table S1. Real-time quantitative PCR reaction was performed on the LightCycler 480 system II (BIOTECON Diagnostics, Roche, Switzerland) and comprised 10 μl of SYBR Premix Ex Taq™ II (Takara Bio, Dalian, China), 0.4 μM of each primer, 2 μl of 1:4 dilution of cDNA and 6.4 μl of ddH_2_O in a final 20 μl volume. The PCR program consisted of a 30 s incubation at 95 °C, followed by 40 cycles of denaturation at 95 °C for 5 s and annealing/elongation at 60 °C for 30 s. A melting curve (from 60 to 95 °C) was run to detect the specificity of the PCR amplification. Each PCR was run in triplicate using cDNAs sourced from three repeatable biological samples per treatment and included no template controls. Maize ubiquitin gene (accession NM001154981) provided an internal standard. The 2^–ΔΔCt^ method was used to quantify the relative abundance of transcripts.

### Ultrastructural observation

Leaf segments (1–2 mm, three plants per treatment) were cut from the second fully expanded leaf at harvest and fixed with 4% glutaraldehyde in 0.2 M phosphate buffer (pH 7.2) at room temperature [61]. Samples were washed three times in 0.1 M phosphate buffer (pH 6.8) and post-fixed in 1% (w/v) OsO_4_, and subsequently dehydrated in an ascending ethanol series. This was followed by infiltration with a series of resin (LR white) mixtures with ethanol, and finally embedded in 100% resin. Ultrasections (80–90 nm) were picked up on copper grids, stained with uranyl acetate for 8 min and washed for 10 min. Then the sections were immediately stained with lead citrate for 5 min, washed as above and air-dried. Images were observed and photographed in a transmission electron microscope (HT7700, HITACHI, Japan).

### Statistical analysis

Data were subjected to ANOVA of General Linear Model (GLM) multivariate analysis with three factors (maize genotype, salt, inoculation treatment) and their interactions as sources of variation, followed by post-hoc multiple comparisons (Tukey’s test) in SPSS 17.0 (IBM, USA). Differences among treatment means were estimated with *P* ≤ 0.05 as the significant level.

## Supplementary Information

**Additional file 1: Table S1.** Gene-specific primers used for quantitative real-time PCR. **Figure S1.** Maize plants of genotypes JD52 (a) and FSY1 (b) inoculated with arbuscular mycorrhizal fungus (*Funneliformis mosseae*) (AM) or without inoculation (NM) in 0 and 100 mM NaCl treatments assessed 59 days after sowing (DAS). Bar = 10 cm

## Data Availability

All data generated or analyzed during this study are included in this published article and its supplementary information files.

## Abbreviations

AM: Arbuscular mycorrhiza
SOS1: Salt overly sensitive 1
HKT1: High-affinity K^+^ transporter 1
NHX: Na^+^/H^+^ exchanger
SKOR: Stelar K^+^ outward rectifier
