# Supplementary material for: Arbuscular mycorrhizal symbioses alleviating salt stress in maize is associated with a decline in root-to-leaf gradient of Na+/K+ ratio
Source: BMC Plant Biol. 2021 Oct 7;21:457. doi: 10.1186/s12870-021-03237-6 (PMC8499542; doi:10.1186/s12870-021-03237-6)
Supplement: Supplementary file 1 — Additional file 1: Table S1. Gene-specific primers used for quantitative real-time PCR. Figure S1. Maize plants of genotypes JD52 (a) and FSY1 (b) inoculated with arbuscular mycorrhizal fungus (Funneliformis mosseae) (AM) or without inoculation (NM) in 0 and 100 mM NaCl treatments assessed 59 days after sowing (DAS). Bar = 10 cm [file 12870_2021_3237_MOESM1_ESM.docx]

**Table S1** Gene-specific primers used for quantitative real-time PCR

| Gene name | Accession | Primer sequence | Product size (bp) | Annealing temperature (°C) |
| --- | --- | --- | --- | --- |
| ZmUBQ | NM_001154981.2 | Forward GTTGAAGCTGCTGCTGTATCTGG | 160 | 58 |
|  |  | Reverse GCGGTCGCACGATAGTTTTG |  |  |
| ZmSOS1 | XM_008647521.3 | Forward ACTTGCAGGAGGAATACAAC | 156 | 58 |
|  |  | Reverse CGAGAAGAGAAGACCACATC |  |  |
| ZmHKT1 | XM_008674901.3 | Forward TGCTAATGTTTATCGTGCTG | 124 | 58 |
|  |  | Reverse AGGCTGATCCTCTTCCTAAC |  |  |
| ZmNHX | NM_001320827.1 | Forward CGTGATGTCGCATTACACCT | 153 | 58 |
|  |  | Reverse CTGGCAAACTCCCACTTCTC |  |  |
| ZmSKOR | NM_001357855.1 | Forward GCAGCACTGAACCTAGAAG | 129 | 58 |
|  |  | Reverse CGTCCTCTGGTCGTAGTT |  |  |

**Fig. S1** Maize plants of genotypes JD52 (a) and FSY1 (b) inoculated with arbuscular mycorrhizal fungus (*Funneliformis mosseae*) (AM) or without inoculation (NM) in 0 and 100 mM NaCl treatments assessed at 59 days after sowing. Bar=10 cm.

**100 mM**

**NaCl**

**AM**

**100 mM**

**NaCl**

**NM**


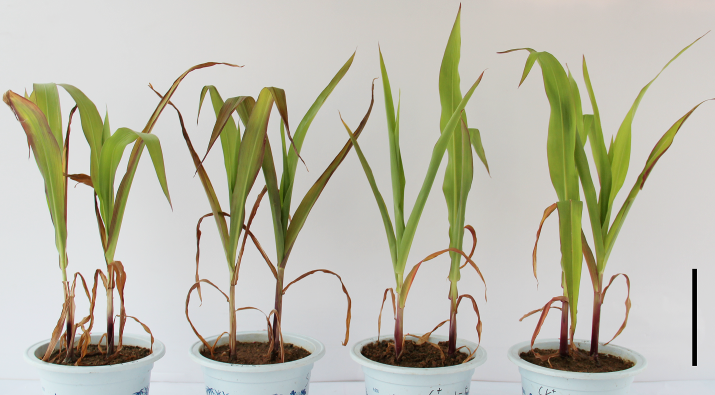

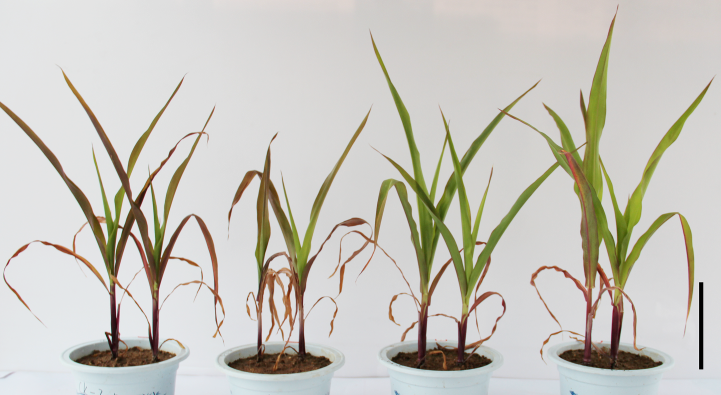


**(b)**

**0 mM**

**NaCl**

**NM**

**0 mM**

**NaCl**

**AM**

**(a)**
